# Theoretical conceptualization of online privacy-related decision making – Introducing the tripartite self-disclosure decision model

**DOI:** 10.3389/fpsyg.2022.996512

**Published:** 2022-10-19

**Authors:** Sina Ostendorf, Matthias Brand

**Affiliations:** ^1^General Psychology: Cognition and Center for Behavioral Addiction Research (CeBAR), University of Duisburg-Essen, Duisburg, Germany; ^2^Erwin L. Hahn Institute for Magnetic Resonance Imaging, Essen, Germany

**Keywords:** self-disclosure, social media, social networks, privacy, decision making, dual-process theory, tripartite model

## Abstract

Self-disclosures on online social networks have received increased attention in the last two decades. Researchers from different disciplines investigated manifold influencing variables, and studies applied different theories to explain why many users share very sensitive and personal information despite potential risks and negative consequences, whereas others do not. Oftentimes, it is argued that self-disclosure decisions result from a kind of rational “calculus” of risks and benefits. However, such an assumption of rationality can and has been criticized. Nevertheless, fundamental cognitive and affective mechanisms that underlie self-disclosure decision making on social networks are still under-explored. By building upon previous self-disclosure theories and models, dual-and tripartite-system perspectives of decision making, and former empirical findings, we propose a Tripartite Self-Disclosure Decision (TSDD) model that conceptualizes inner processes of online self-disclosure decision making. Central to this model is the proposed interaction of three neural and cognitive/affective systems: a reflective, an impulsive, and an interoceptive system. We further highlight individual and environmental features, which can impact individuals’ online self-disclosure decisions by (interactively) influencing the proposed inner decision-making processes targeting the aforementioned three systems. Possible short- and long-term consequences are also discussed, which in turn can affect certain model components in subsequent self-disclosure decision situations. By taking such a neurocognitive perspective, we expand current research and models, which helps to better understand potentially risky information sharing on online social networks and can support attempts to prevent users from incautious self-disclosures.

## Introduction

Around the world, a large number of people from diverse backgrounds regularly use online social networks such as Facebook, Instagram, or Twitter. In just 13 years after its launch in 2004, Facebook has surpassed two billion active users and reports around 2.93 billion monthly active users in the first quarter of 2022 (Statista, 2022a). Instagram and Twitter follow with two billion and 330 million monthly active users, respectively (Statista, 2022b, 2022c). In addition to these ‘Western applications’, a big competitor is China’s Sina Weibo, with 582 million monthly active users in the first quarter of 2022 (Statista, 2022d). Such platforms have three key aspects in common: they enable their users to create unique profiles and individual content available for others, they allow to articulate connections via friends lists, and enable to consume and interact with content of online connections (Ellison and Boyd, 2013). Social networks provide many tools with which individuals can fulfill their fundamental need for social connection and belonging and they enable the experience of immense gratification (e.g., Huang et al., 2014; Ostendorf et al., 2020). By disclosing personal information via profile, posts, or stories, individuals can maintain and strengthen their friendships and relationships, they can receive social support, build new social bonds, present themselves, and increase social capital (e.g., Krasnova et al., 2010; Krämer and Haferkamp, 2011; Ellison and Boyd, 2013; Liu and Brown, 2014; Taddicken, 2014; Cheung et al., 2015; Masur, 2018; Lu and Lin, 2022).

However, although many benefits are associated with the use of online social networks, potential negative aspects have received attention as well. Due to the ubiquity of such services, individuals can, for instance, perceive an increased pressure to always be available to everyone (e.g., Vorderer et al., 2016) or experience a reduced well-being, especially when passively using social networks (e.g., Verduyn et al., 2021). A growing body of research also addresses a potential loss of privacy associated with the use of these platforms. Social networks such as Facebook or Instagram do not provide their users with unlimited control over their shared information (Marwick and Boyd, 2014; Trepte, 2021), which can cause negative and undesired consequences of varying severity, especially since access to and aggregation of data can be very easy (Barth and de Jong, 2017). On a horizontal level, individual privacy can be violated if personal information is undesirably accessed or used by other members (including friends and co-workers, but also mere acquaintances and strangers). On a vertical level, it can be violated if the platform itself or third parties misuse the shared content (see Bartsch and Dienlin, 2016; Quinn and Epstein, 2018). Overall, negative consequences due to self-disclosures can occur in both the short- and long-term, reaching from immediate negative feedback from others to sexual online harassment and hostility up to privacy intrusions such as commercial/criminal exploitation or identity theft (e.g., Debatin et al., 2009; Walrave et al., 2012; Aharony, 2016).

However, even if users seem to be concerned about their privacy, a number of studies did not find that this is consistently associated with adequate privacy-related behaviors including reduced information disclosures (e.g., Acquisti and Gross, 2006; Tufekci, 2008; Zafeiropoulou, 2014). This gap between concerns and actual behaviors is also known as *privacy paradox* (e.g., Barnes, 2006; Aivazpour et al., 2017; Barth and de Jong, 2017; Kokolakis, 2017). In order to explain why such seemingly paradoxical behaviors occur, researchers take different perspectives for their investigations (e.g., a social psychological or information science perspective). However, in many studies, researchers apply theories and models that cover a quite rational perspective and do not comprehensively include impulsive, intuitive, or even interoceptive processes. By taking a rational choice approach, it is argued that individuals weigh potential risks and benefits and decide to share information if the expected benefits exceed potential risks (e.g., Debatin et al., 2009; Lee et al., 2013; Li et al., 2016). This indicates that individuals are rational decision makers when deciding to self-disclose on social networks, which, however, seems not to be a sufficient explanation for many self-disclosure decision situations. Especially on social networks, risks can be abstract (e.g., privacy violations such as identity theft) and may not always be fully comprehended or processed (e.g., Masur, 2018). Hence, different authors also investigate cognitive biases, heuristics, and affective processes that can influence individuals’ privacy-related decisions online (e.g., Acquisti and Grossklags, 2007; Sundar et al., 2013; Acquisti et al., 2015; Yu et al., 2015; Kehr et al., 2015a). Researchers highlighted that approaches which assume that individuals (consistently) engage in deliberate analysis when it comes to privacy-related online decisions (i.e., engage in high-effort processing) overlook the probable involvement of low-effort processes which are based on, for instance, affect or heuristics, and they hence directed the focus to such processes (e.g., Lowry et al., 2012; Dinev et al., 2015).

Nevertheless, the fundamental cognitive and affective mechanisms that underlie individuals’ self-disclosure decisions on social networks and that may explain behavioral differences between individuals are still under-explored. From a theoretical standpoint, potentially involved inner processes beyond reflective ones and their interplay during self-disclosure decision making need closer examination. Since focusing on a rational “calculus” appears not to be a sufficient explanation for individual’s self-disclosure decisions on social networks, more systematic research on involved intuitive, impulsive, as well as interoceptive processes would enrich research in this area. The purpose of the present paper therefore is to address this theoretical gap. We propose a theoretical framework that illustrates expected inner processes of self-disclosure decision making on social networks by including a tripartite proposition of involved (and interacting) neural systems. Building upon dual-and tripartite-process models of decision making, we elucidate the assumption that the decision to self-disclose can also result from more impulsive and short-term oriented compared to reflective and long-term oriented decision making, which may be amplified by interoceptive processes. Furthermore, we distinguish between and exemplify individual and environmental features that both can (individually and in interaction with each other) influence the inner decision-making processes that form the final decision. Lastly, possible consequences are differentiated with regard to their temporal character and positivity/negativity. By taking a neurocognitive perspective, our tripartite model helps to advance the understanding of why so many individuals provide very sensitive and personal information despite possible privacy violations and other negative consequences, whereas other users do not. We suggest that not only reflective processes can be involved, but that impulsive and intuitive as well as interoceptive processes may also play a crucial role, which helps to complete the picture. We further aim at guiding research in deriving systematic hypotheses on the interplay of different factors predicting self-disclosure decisions and suppose that our theoretical propositions can also help to improve technical options to support individual’s self-disclosure decision making on social networks.

In the following, we present prior theoretical assumptions and models which have oftentimes been applied to explain various online privacy-related decisions including self-disclosure decisions on social networks. We outline that a compelling account of how individuals come to different self-disclosure decisions with a focus on inner processes is still lacking. Subsequently, we present key assumptions of dual-and tripartite-process theories of decision making, which serve as basis for our theoretical framework that is presented in detail afterwards. Finally, we discuss our propositions and provide future directions, followed by concluding remarks.

### Theoretical assumptions and models previously used to explain online privacy-related decision making

Different literature reviews (see Barth and de Jong, 2017; Kokolakis, 2017; Gerber et al., 2018) have been published outlining that many investigations on information disclosures in different online contexts including social networks focus on the idea of rather rational or reflective calculations, which is grounded in different theories (often addressing offline contexts) such as the *Expectancy Theory* (Vroom, 1964), the *Protection Motivation Theory* (Rogers, 1975), the *Theory of Reasoned Action* (Ajzen and Fishbein, 1980), the *Theory of Planned Behavior* (Ajzen, 1985), or the *Privacy Calculus Theory* (Culnan and Armstrong, 1999). For instance, according to the latter, individuals are expected to perform a kind of risk–benefit calculation on a rational level as a basis for their behaviors. Applied to self-disclosure decision making on social networks, it is assumed that users weigh the risks and benefits of sharing personal information and engage in self-disclosure if the expected gains outweigh observed potential negative consequences (e.g., Debatin et al., 2009; Lee et al., 2013; Li et al., 2016). Yet, many studies in the field of online privacy and information disclosure have included such theories (e.g., Dinev and Hart, 2006; Krasnova et al., 2010; Xu et al., 2011; Yao, 2011; Dienlin and Trepte, 2015; Dienlin and Metzger, 2016).

However, assuming that there is always a rational weighing of benefits and risks can be challenged, since especially long-term negative consequences (e.g., undesired commercial or criminal use of shared information) are likely more abstract and less salient compared to possible short-term rewards (e.g., immediate gratification due to Likes). This results from the fact that social networks do not provide thorough information about corresponding risks (Taddicken and Jers, 2011; Efroni et al., 2019). Consequently, this may complicate their evaluation (as part of individual’s inner decision-making processes) and possibly leads to more impulsive or intuitive rather than reflective decisions (Ostendorf et al., 2020). The assumption of limited rational processes is also represented in other theoretical approaches used to explain privacy-related online behaviors (see also Barth and de Jong, 2017; Kokolakis, 2017; Gerber et al., 2018). For example, different works refer to the notion of *incomplete information* when investigating online information sharing, which goes along with further aspects such as over−/underestimation of negative outcomes and benefits (e.g., Acquisti and Grossklags, 2005; Flender and Müller, 2012). Besides, the *Theory of Bounded Rationality* (Simon, 1982), a well-known concept in cognitive and social psychology, has also been applied to online information sharing (e.g., Acquisti and Grossklags, 2005; Wang and Yan, 2017). This theory proposes that human beings do not have full access to all relevant information, that they are cognitively limited, and also do not always have sufficient time to make their decisions in a fully rational manner (see Simon, 1982), which is also applicable to online self-disclosure decision situations. Moreover, *cognitive biases* and *heuristics* (e.g., *optimism bias*) have also been investigated in different publications (e.g., Acquisti and Grossklags, 2003; Cho et al., 2010; Gambino et al., 2016; see also Irwin, 1953; Slovic et al., 2002). However, research on non-reflective processes still needs to increase, which especially applies to the investigation of interoceptive processes. Studies on the role of interoceptive processes in the context of social networks are to date very scarce, but appear important for a deeper understanding of individuals’ online self-disclosure decisions (see section 1.2).

Besides those approaches, there are also a few valuable theoretical models specifically focusing on the concepts of self-disclosure and privacy (offline and online; see also Masur, 2018). The *Disclosure Decision Model* by Omarzu (2000), for example, is a process model that incorporates situational cues and individual differences, which are expected to shape the salience of specific (social) goals, as well as three stages of self-disclosure decision making (“entering the situation,” “selecting a strategy and searching for targets,” and “subjective utility versus subjective risk,” pp. 178/179). If one or more (potentially overlapping or even conflicting) goals are salient in stage one, an individual is expected to evaluate in stage two whether self-disclosure would be an appropriate strategy or not (i.e., to reach the goal) and whether a suitable target is available or not (i.e., desired recipients). If both applies, individuals are assumed to enter the third stage in which they decide how much, how long, how intimately, and how broadly they self-disclose. Thereby, the model proposes that there is a weighing of the subjective utility of possible benefits and the subjective probability of risks.

Another framework is the *Disclosure Processes Model* by Chaudoir and Fisher (2010). This model importantly highlights the role of possible long-term outcomes and resulting feedback loops that should not be left behind when conceptualizing individuals’ self-disclosure decision making on social networks. Besides the consideration of antecedent goals, the resulting disclosure event, and factors mediating between the disclosure event and long-term outcomes, it accentuates the potential influence of long-term outcomes on future disclosure decisions in terms of “upward spirals toward greater visibility” versus “downward spirals toward greater concealment” (Chaudoir and Fisher, 2010, p. 250). This view of reinforcing mechanisms and feedback is also well known in other theoretical frameworks of risky decision making (e.g., Brand et al., 2006; Schiebener and Brand, 2015).

Besides, the procedural *Privacy Process Model* by Dienlin (2014) is placing special emphasis on the concept of privacy. The privacy context (denoted as being objective) is proposed to be perceived subjectively by an individual and this perception is expected to subsequently influence the respective behavioral decision, namely self-disclosure. Further, as part of an evaluation process, individual’s privacy perception and behavior are assumed to be constantly compared to a desired status, whereby a privacy regulation (with regard to the context or the behavior) is taking place if there is an imbalance between the current and the desired status.

Masur (2018) further introduced his view of *Situational Privacy and Self-Disclosure*. Closely related to Omarzu (2000), he (even stronger) argues that situational circumstances play an important role in the context of self-disclosure. The model presents three procedural stages: “(1) pre-situational privacy regulation processes, (2) situational privacy and self-disclosure processes, and (3) post-situational evaluation processes” (Masur, 2018, p. 177), with a central role of the second stage. It is argued that an individual’s self-disclosure extent depends on different personal and environmental features and their interplay. Especially interactions between non-stable personal features and environmental features in a given situation are expected to shape individuals’ self-disclosures, whereby this is further influenced by trait and trait-like personal features. Subsequently, post-situational evaluation processes (e.g., regarding the efficiency of self-disclosure) are expected to occur. Further, in the pre-situational stage, individuals potentially choose and manipulate the respective environment to achieve a desired privacy level before engaging in self-disclosure.

In summary, the aforementioned models already provide important theoretical and process-oriented assumptions. Important distinctions are made between individual characteristics, situational/context-related factors, and specific decision-making steps (see *Disclosure Decision Model* by Omarzu, 2000; *Situational Privacy and Self-Disclosure* by Masur, 2018). Further, the role of possible (long-term) outcomes and their potential reinforcing effects with regard to subsequent decision situations (see *Disclosure Processes Model* by Chaudoir and Fisher, 2010) and the importance of perception processes and possible privacy regulations in the context of online self-disclosures are highlighted (see *Privacy Process Model* by Dienlin, 2014; Masur, 2018). However, besides that it is sometimes quite vague what is actually meant by individual differences and situational cues (e.g., in the *Disclosure Decision Model* by Omarzu, 2000, as also mentioned by Masur, 2018), these frameworks take the perspective of rather strategic self-disclosure decisions and are especially lacking the explicit consideration of underlying non-reflective processes. As highlighted above, processes beyond reflective ones need to be considered for human decision making and thus also for self-disclosure decisions on social networks. From a neurocognitive perspective, it is hence of great importance to provide a compelling account of psychological mechanisms underlying self-disclosure decisions on social networks. However, a theoretical framework that explicitly incorporates potentially involved inner processes and their interplay, and that broadens the perspective by including reflective as well as intuitive/impulsive and interoceptive processes, is still lacking. The aim of the current paper is to fill this gap. Dual-and tripartite-process theories, which focus on processes that are at the core of human decision making, will thereby serve as a basis for our propositions (see next section).

### Dual- and tripartite-process theories of decision making

The literature in the fields of (neuro-)cognitive and social psychology holds many dual-process theories with different foci and perspectives (for overviews see Evans, 2008; Evans and Stanovich, 2013). Although different perspectives exist, there is a common idea which brings those different approaches together: It is expected that two different forms of processing are in place that shape human reasoning and decision making (e.g., Stanovich, 1999; Kahneman and Frederick, 2002; Evans, 2003, 2006; Strack and Deutsch, 2004; Kahneman, 2011; Schiebener and Brand, 2015). In more detail, it is assumed that processes of, for instance, intuitive, emotional, or impulsive kind, as well as processes of, for instance, strategic, reflective, or analytical kind can be involved when making a decision (e.g., Kahneman, 2011; Schiebener and Brand, 2015). In this paper, we follow the idea that these two forms of processing involve two neural systems that are not strictly separated from each other but rather interact with one another when forming the final decision (e.g., Kahneman, 2011; Schiebener and Brand, 2015). The reflective system is considered to function slowly, serially, and cognitively-controlled, and it is also known as system 2 or rational-analytical system (e.g., Epstein et al., 1996; Stanovich, 1999; Kahneman, 2003, 2011). The impulsive system is considered as a fast and parallel processing system (also termed system 1 or intuitive-experiential system) that draws on past experiences and emotions, and only needs low effort (e.g., Epstein et al., 1996; Kahneman, 2003). It is also assumed that immediate gratification (or punishment) is processed via the impulsive system and that cognitive control over impulsive responses in order to reach higher long-term goals is enabled by the reflective system (see also Bechara, 2005). Thus, if information is processed via the impulsive system, it is likely accompanied with emotional reactions or somatic activity (e.g., increased heart rate), while a reflective information processing involves executive functions and working memory (see Evans, 2008; Schiebener and Brand, 2015). On a neural level, the impulsive system involves structures associated with the limbic-ventral striatal loop, such as the ventral striatum and the amygdala, and the reflective system involves structures associated with the prefrontal-dorsal striatal loop, such as the dorsolateral prefrontal cortex (Rogers et al., 1999; Labudda et al., 2008; Kühn et al., 2011; Schiebener and Brand, 2015). Further, it is assumed that both systems can be active in parallel and can interact with each other during decision making, whereby one of the systems will act as the leading mode (Schiebener and Brand, 2015). The balance between these two neural systems is thus essential for the shortsightedness of the respective decision. Moreover, Schiebener and Brand (2015), for example, point out that individual, environmental, and situational features influence the inner decision-making processes and thus trigger a predominant system. This view of different inner processes being involved in human decision making hence appears important to both better understand why individuals provide much personal information despite potential risks and how they can be supported to improve their decision making.

However, neurocognitive research on decision making is not limited to dual-process approaches, but recently also focuses on a tripartite perspective. This perspective suggests that a third system – covering interoceptive awareness – can alter the balance between the reflective and the impulsive system (Wood and Bechara, 2014). On a neural level, previous research found that the interoceptive system is mainly associated with the insula, which is expected to translate somatic states into more conscious states of mind (Noël et al., 2013). Xue et al. (2010) further found that the activation of the insula during decision making was associated with the personality trait of urgency and influenced the extent of subsequent risky decisions. They concluded that “the insula plays an important role in activating representations of homeostatic states associated with the experience of risk, which in turn exerts an influence on subsequent decisions” (Xue et al., 2010, p. 709). This highlights the additional relevance of an interoceptive system for human decision making. Other research in different contexts also focused on a tripartite approach. For instance, the role of the insula in modulating the balance between the impulsive system and the reflective system has been highlighted and examined in the context of problematic eating and when facing tempting food cues (see Chen et al., 2018; He et al., 2019). Wei et al. (2017) further proposed a tripartite neurocognitive model of Internet Gaming Disorder and Turel et al. (2021) found that gamers who were deprived of gaming showed an increased activation of the left insula when being exposed to video gaming cues versus neutral cues. Further, left insula activation was also positively associated with left ventral striatum activation and negatively with left dorsolateral prefrontal cortex activation, supporting the tripartite perspective (Turel et al., 2021). Based on research on problematic and addictive behaviors (e.g., Naqvi et al., 2007; Noël et al., 2013), another study by Turel and Bechara (2016) applied assumptions regarding the role of interoceptive processes to the area of general and impulsive information technology use. They investigated a tripartite model with regard to the extent of social networks use (in terms of duration and frequency) and impulsive social networks use. They found that temptations (representing the interoceptive system) strengthened the effect of habit (representing the impulsive system) on the extent of social networks use as well as on impulsive social networks use, while temptations reduced the effect of satisfaction and behavioral expectations (standing for the reflective system) on both the extent and impulsiveness of social networks use. These findings give important indications that not only an impulsive and a reflective system can guide human behavior and decision making, but also an interoceptive system. As stated by the authors, “technology use behavior, […], can also often be influenced by salient situational temptations, which violate the balance between users’ reflective and impulsive information processing processes” (Turel and Bechara, 2016, p. 7). Consequently, body physiology and visceral status may also play an important role in the context of online self-disclosures. Following the important conceptualizations of dual-and tripartite-process models of decision making, we believe that it is also necessary for research on online self-disclosure to extent the frequently proposed perspective of a rather rational decision maker and to provide an integrative framework that additionally includes intuitive/impulsive as well as interoceptive processes.

## The tripartite self-disclosure decision (TSDD) model

**Figure 1:**
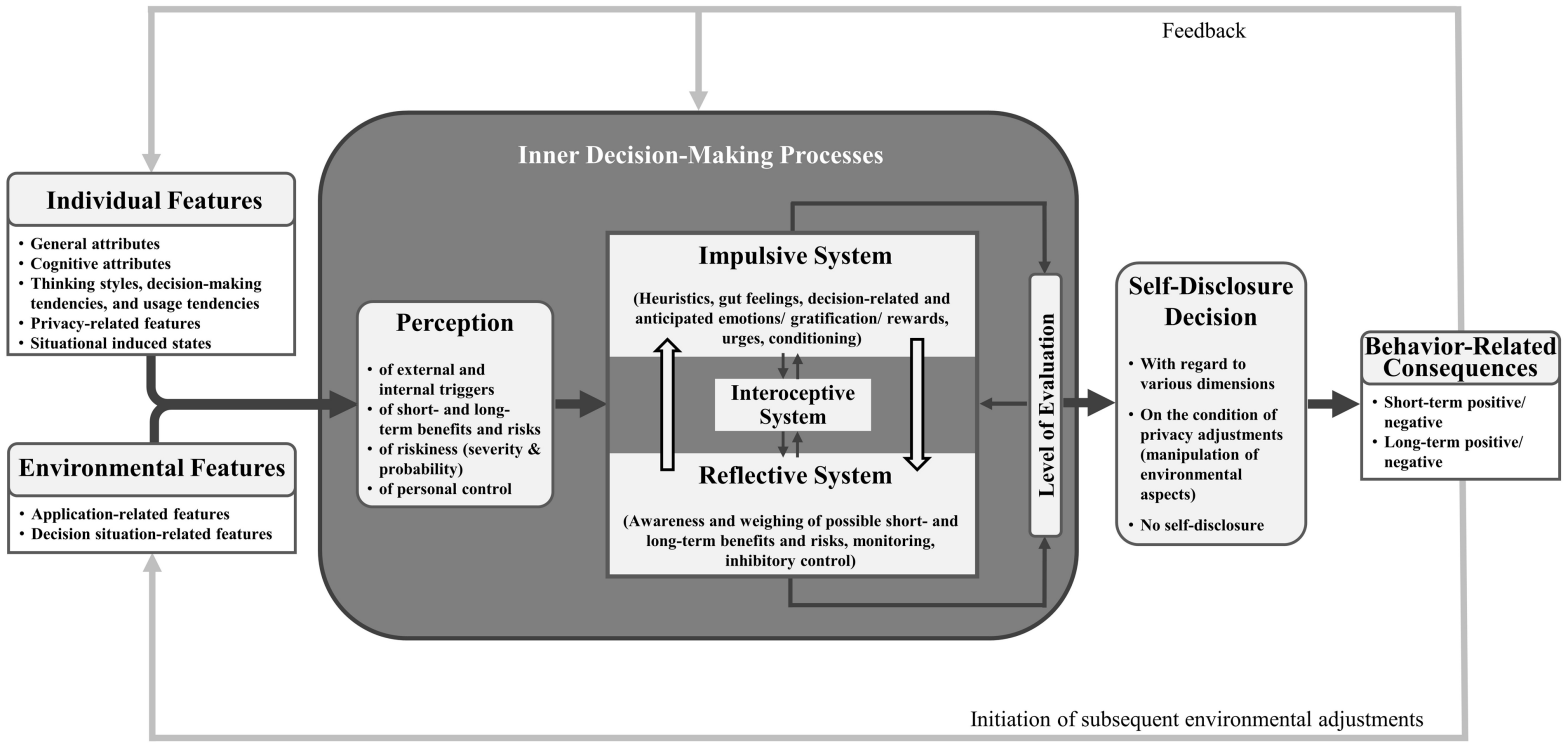
The Tripartite Self-Disclosure Decision (TSDD) model with bold arrows representing the main pathway of the self-disclosure decision-making process on social networks.

We now propose the Tripartite Self-Disclosure Decision (TSDD) model, which integrates previous assumptions from both several self-disclosure theories and general decision-making models. It conceptualizes the respective components and inner processes expected to be involved in the decision to self-disclose (or not to self-disclose) on social networks. The main components of the TSDD model are *individual and environmental features*, *inner decision-making processes*, the *self-disclosure decision* stage, *behavior-related consequences*, and *feedback loops*. The main assumption of this model is that different individual and environmental features influence individual’s inner decision-making processes including the interplay of three neural systems (an impulsive, reflective, and interoceptive system), which shapes evaluation processes that lead to the final self-disclosure decision, followed by various consequences. Based on this proposed general connection between the model’s components, we highlight that for many self-disclosure decisions on social networks, especially the impulsive (and interoceptive) system may play a predominant role due to specific individual (e.g., situational needs, trait impulsivity) and environmental features (e.g., push-notifications of social networks). However, the reflective system may also be triggered by protective features (e.g., the need for privacy or a warning message hinting at potential negative consequences), which could lead to more deliberate self-disclosure decisions that reduce the risk of experiencing negative consequences (e.g., increased hostility or privacy intrusions). The model is illustrated in Figure 1. In the following, we explain all components in more detail and highlight corresponding and related previous empirical findings.

### Individual and environmental features

**Figure 2:**
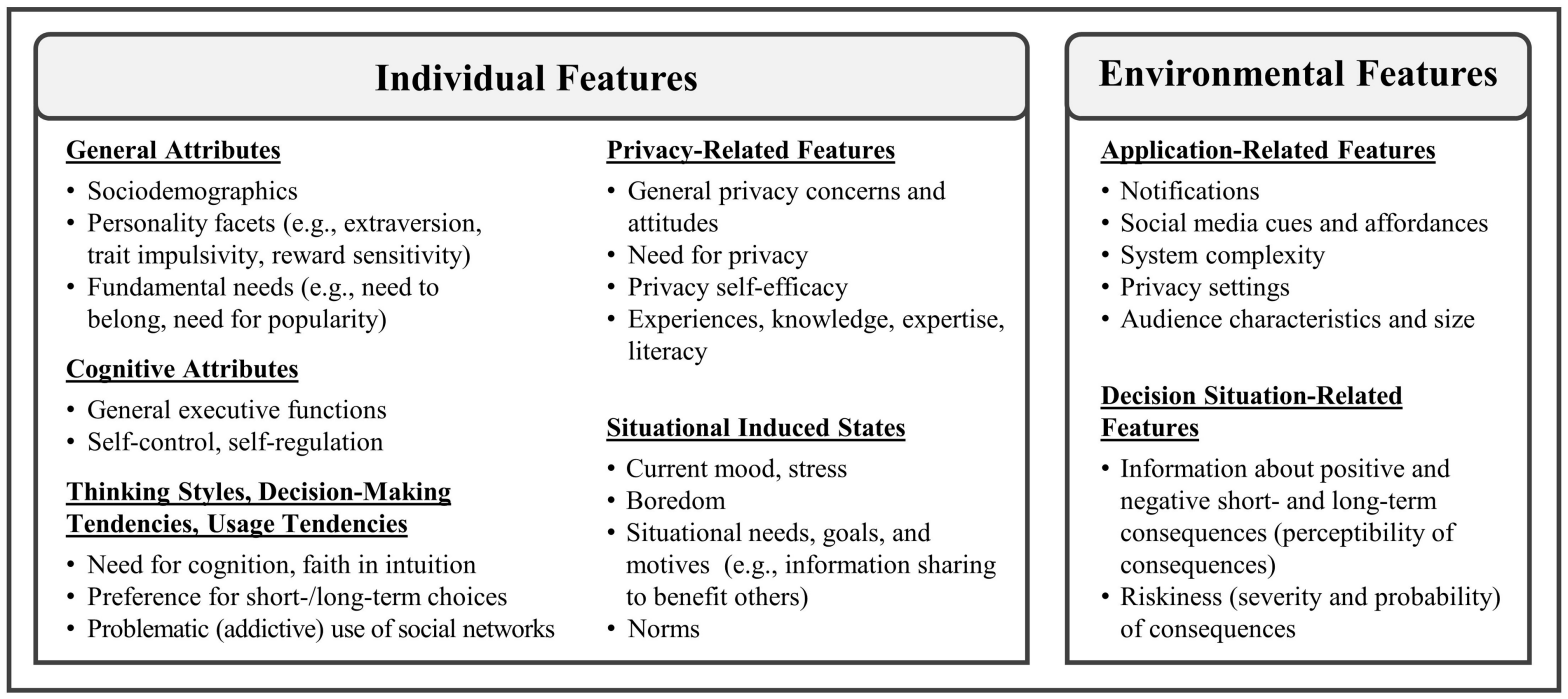
Selection of individual and environmental features relevant for self-disclosure decision making on social networks.

In line with Schiebener and Brand (2015), Masur (2018), and Omarzu (2000), the first component of the TSDD model consists of *individual and environmental features* (see Figure 2). Previous research has investigated manifold variables and their relations and contributions to individuals’ online self-disclosure decisions. With respect to our theoretical process model, we try to cover many of these, but do not intend to be exhaustive.

#### Individual features

Individual features include stable and non-stable aspects that are attributed to the person in a certain self-disclosure decision situation. Categories in which we have arranged the aspects are (i) *general attributes*, (ii) *cognitive attributes*, (iii) *thinking styles, decision-making tendencies, and usage tendencies*, (iv) *privacy-related features*, and (v) *situational induced states* (see Figure 2).

Under the term *general attributes*, we subsume sociodemographic characteristics, personality facets (e.g., extraversion, trait impulsivity, or reward sensitivity), and general/fundamental needs (e.g., the need to belong). Following previous studies, younger individuals were often found to share more information on social networks than older people (e.g., Christofides et al., 2012; Walrave et al., 2012) and their shared information was mentioned to be growing (Madden et al., 2013). Gender was in general found to be a rather weak predictor, whereby women appear to disclose more broadly and in greater depth than men (see Gerber et al., 2018). However, there are also studies reporting men to disclose more demographic details on Facebook (Aharony, 2016) and more basic and contact information (Special and Li-Barber, 2012) compared to women. Personality characteristics including extraversion or narcissism were related to the disclosure of different kinds of information within Facebook status updates (Marshall et al., 2015). Furthermore, a recent study reported motor impulsivity to be positively related to individual’s self-reported information disclosure and to moderate the relation between privacy concerns and information disclosure (Aivazpour and Rao, 2020), which may indicate the involvement of non-reflective inner processes. In line with possible impacts of different impulsivity facets, neurocognitive studies highlighted the role of individual’s reward sensitivity in the context of Likes on social networks (Sherman et al., 2016, 2018) and reported associations with a problematic (addictive) use of the Internet (He et al., 2017), which in turn can lead to higher self-disclosure levels (e.g., Molavi et al., 2018, see category *thinking styles, decision-making tendencies, and usage tendencies*). Regarding individual’s general needs, the need for popularity was shown to impact different social networks usage behaviors including strategic self-presentation, profile enhancement, and the disclosure of feelings (Utz et al., 2012), and higher levels of need to belong were positively associated with the depth of self-disclosures (more intimate information) in status updates (Winter et al., 2014).

Consistent with neurocognitive models of decision making (e.g., Schiebener and Brand, 2015), the category *cognitive attributes* encompasses for example general executive functions, working memory, and the ability to control and regulate oneself, which are important for reflective functioning. Muscanell (2013), for instance, demonstrated that trait self-control can negatively predict the disclosure of self-damaging information (e.g., regarding alcohol consumption) on social networks. Molavi et al. (2018) moreover reported that self-regulation was negatively associated with what they called ‘toxic self-disclosure’, covering riskier self-disclosures (with regard to a specific culture) compared to only the breadth and depth of disclosure. In an experimental study, Veltri and Ivchenko (2017) further investigated the impact of induced cognitive scarcity in the form of ego depletion and cognitive load on the disclosure of sensitive information. They found that both had a significant positive effect on the amount of disclosed information, whereby working memory load was associated with a slightly higher level of information disclosure compared to the ego depletion condition.

Within the category *thinking styles, decision-making tendencies, and usage tendencies*, we refer, for example, to the need for cognition and faith in intuition (indicating a preference for reflective or intuitive processing, respectively), which were highlighted by Kehr et al. (2015b) to be differently associated with the thoroughness of weighing risks and benefits of information disclosure. They found that individuals with the tendency for experiential thinking seem to overleap rational considerations and, in contrast, individuals scoring high on rational thinking seem to reflect more on risks and benefits. These results are in line with formerly mentioned dual-process theories (Bechara, 2005; Schiebener and Brand, 2015) and underline that the *Privacy Calculus* framework might be a useful explanatory approach under specific circumstances, but that it is likely not sufficient to be generally applied. Following this, individual’s general decision-making tendencies (e.g., the preference for short-term over long-term choices) constitutes another important individual feature. A recent study highlighted that individual’s preference for choosing short-term rewarding options while neglecting long-term (mainly negative) outcomes is associated with an increased extent of self-disclosure within posts on Facebook (Ostendorf et al., 2020). In this study, it was also found that specific usage tendencies, namely problematic (addictive) social-networks-use tendencies, were related to higher levels of self-disclosure within posts, which overall provides indications of involved impulsive processes (see Ostendorf et al., 2020). In line with this, a problematic (addictive) use of the Internet in general was also found to be significantly associated with higher levels of online self-disclosure (Molavi et al., 2018).

*Privacy-related features* that can influence online self-disclosure decisions are, for example, general privacy-related attitudes and concerns, privacy self-efficacy, individual’s need for privacy, prior experiences, and literacy including knowledge and specific skills. Many studies already investigated the role of concerns and attitudes but found mixed results. Some studies highlighted a direct relation to online self-disclosure and privacy-management behaviors (e.g., Dwyer et al., 2007; Wu et al., 2012; Kezer et al., 2016), whereas many others did not find a stable association (e.g., Acquisti and Gross, 2006; Tufekci, 2008; Reynolds et al., 2011; Zafeiropoulou, 2014; Quan-Haase and Elueze, 2018) or specifically investigated the interplay of concerns and other factors in the prediction of privacy-related online behaviors, such as self-efficacy in privacy management, perceived social relevance of social web applications, or impulsivity facets (e.g., Taddicken, 2014; Chen and Chen, 2015; Aivazpour and Rao, 2020). These results again emphasize the need for a better understanding of psychological processes underlying privacy-related decisions. Regarding the need for privacy, Trepte and Masur (2020) argued that it “may be a buffering factor against unhealthy and unsecure uses of online communication” (p. 3), which would also include incautious self-disclosures on social networks. Further, prior experiences with privacy invasions, (declarative and procedural) knowledge, and skills can also act as protective factors (for example by translating into privacy-management behaviors), which was already highlighted by authors focusing on different online disclosures (e.g., Debatin et al., 2009; Bansal et al., 2010; Park, 2013; Bartsch and Dienlin, 2016; Kezer et al., 2016; Büchi et al., 2017).

Besides trait or trait-like features, specific *situational induced states* (see also Schiebener and Brand, 2015; Masur et al., 2018) are also considered. For example, individual’s current mood (e.g., excitement), stress level, further states of mind (e.g., boredom), situational needs, motives, and goals, or norms can influence individual’s online self-disclosures. It was, for instance, found that people in a positive mood disclosed more intimate and more positive information during a computer-mediated interaction than those in a negative or neutral mood (Forgas, 2011). Further, arguing from the theoretical perspective of general decision making, perceived stress (also closely related to extreme mood such as depressive state) may affect cognitive processes, for instance by directing individual’s attention to short-term rewarding options and probably risky alternatives, which can in turn result in the respective behavior (see also Starcke and Brand, 2012). Moreover, the experience of boredom may also be an important state of mind (by triggering non-reflective processes), particularly for younger individuals (e.g., Davis, 2012). Thus, situational-specific states and temporary emotional experiences could play an elementary role which needs to be investigated more deeply in future studies. Besides, it was argued that for achieving specific goals and for fulfilling current motives or situational needs (e.g., information sharing to benefit others, managing/maintaining specific relationships), varying depths and breadths of self-disclosure may be required for which differently safe environments may be chosen (e.g., instant messenger chats versus status updates and wall posts; see Bazarova and Choi, 2014; Masur, 2018). Another aspect that can influence self-disclosure decision-making processes on social networks are norms, which are situation- and context-specific and can be differentiated into descriptive and injunctive norms (Cialdini et al., 1991). Zlatolas et al. (2015), for instance, found that privacy social norms, representing the perception that persons close to oneself expect a specific privacy-enhancing behavior (thus injunctive norms), were significantly related to lower levels of self-disclosure on social networks. In a recent study, Ostendorf et al. (2022) further found that being confronted with posts from other users in which much information is shared (i.e., descriptive norms) can lead participants to disclose significantly more than those participants who are confronted with other users’ posts which contain only little personal information, indicating the involvement of intuitive and heuristic processes.

#### Environmental features

Several environmental features can also play an important role for individual’s decision making (e.g., Omarzu, 2000; Schiebener and Brand, 2015; Masur, 2018). Those we consider relevant for users’ self-disclosure decisions on social networks are subsumed under *application-related features* and *decision situation-related features*. With regard to *application-related features*, we include applications’ notifications (push notifications), cues (e.g., application icons), affordances (e.g., Like-buttons, design aspects), the complexity of the respective system, privacy settings as well as audience characteristics and size. Following Trepte (2015), cues and affordances that are familiar to the user (termed warm affordances) stand in contrast to cold affordances (which are sparsely familiar to the user, e.g., the platform’s privacy conditions) in the way that they encourage individuals to provide and share content and are easier accessible than cold affordances. Thus, specific application-related cues and affordances can challenge each other and can thereby influence individuals’ self-disclosure decisions on social networks. Nyshadham and Van Loon (2014) further stated that it can depend on the design of a website whether privacy-related decisions are more likely made by relying on cognitive ease (i.e., effortless intuitive processes) or on cognitive strain (i.e., effortful reflective processes). Moreover, notifications may also constitute an important factor, since they likely affect individuals’ social media use (see Du et al., 2019) and an increased usage of social media applications was in turn found to lead to a greater self-disclosure tendency (Walrave et al., 2012; Chang and Heo, 2014). Another noteworthy aspect that may affect individuals’ self-disclosure decisions on social networks is the structural complexity of the respective platforms. Given that social networks live from the data their users provide them, their information and data dissemination model is quite complex and often not easy to understand. Due to the multitude of integrated third party applications, their growing interconnections, and the way that these connections “are (not) communicated can make it hard to understand and manage how personal information is shared and stored online” (King et al., 2011, p. 3). Thus, this complexity may influence individuals’ self-disclosures by influencing inner decision-making processes, which is outlined in greater depth in the next section. Along with the structural complexity of social networks, the scope of possible privacy settings and the degree of difficulty or intricateness to manage them needs also to be taken into account. Following the study of Shane-Simpson et al. (2018), differences in available privacy settings can create (in combination with other factors such as provided communication modes, privacy concerns, or personality characteristics) preferences for different social networks (i.e., Twitter, Instagram, or Facebook) and can thereby elicit different levels of self-disclosure. Further, the social sphere that is able to access one’s shared information, meaning the audience with its characteristics and size, can impact the extent to which an individual self-discloses. For example, using smaller sub-networks of desired people was found to be associated with increasing self-disclosures since it can strengthen the experience of bonding and bridging social capital (e.g., Stutzman et al., 2012). However, other works also reported that preferring a specific network (i.e., Twitter) was associated with having a public profile rather than a private one (thus having no audience restrictions) and subsequently also high levels of self-disclosure as well as high experienced bridging social capital (Shane-Simpson et al., 2018).

With regard to *decision situation-related features*, explicit information about positive and negative short- and long-term consequences (thus their perceptibility) and information about the riskiness (including severity and probability of risk occurrence; see Rohrmann, 2008) attributable to a specific self-disclosure decision situation can also play an important role. The level of perceptibility is closely related to design and complexity of the respective service and is likely not balanced in terms of positive/negative and short-/long-term consequences, meaning that long-term (negative) consequences are rather intangible compared to short-term (positive) consequences (e.g., Taddicken and Jers, 2011; Efroni et al., 2019). However, providing information on possible negative short- and long-term consequences within a warning message was recently shown to be able to reduce the likelihood that a post is created on a (fictitious) social network, indicating the triggering of reflective processes (Ostendorf et al., 2022). In general, a varying amount or kind of information that is provided in a specific decision-situation (for instance, percentages on the likelihood of specific risks when sharing the phone number with different audiences or the potential severity of specific risks when sharing one’s personal address or religious views) can be relevant for individual’s self-disclosure decision making. Nevertheless, such environmental features and their impact on individuals’ self-disclosure decisions on social networks still need to be investigated in more detail. Moreover, the structure and functionalities of those manifold platforms vary greatly, which can further impact individual’s inner decision-making processes to different extents.

### Inner decision-making processes including a tripartite structure of involved neural systems

After reviewing individual and environmental features that can impact self-disclosure decisions on social networks, one aspect still remains not sufficiently understood: the mechanisms that underlie the decision for or against the disclosure of personal information. In the following, we therefore describe the inner decision-making processes that are assumed to be central to such decisions.

We argue that, based on individual and environmental features, individuals subsequently perceive specific self-disclosure situations differently (see also *Privacy Process Model* by Dienlin, 2014). That means that the *perception* of, for example, specific internal and external triggers (e.g., current mood and notifications), possible short- and long-term benefits and risks (e.g., reduction of negative mood), or one’s personal control (e.g., over personal information) is subjective, which then influences the interplay of the impulsive, reflective, and interoceptive system. In accordance with this notion, an individual who has, for instance, in general a high need to belong and in a specific situation feels socially isolated may perceive this feeling as highly unpleasant and may especially perceive the expected benefits associated with the decision to self-disclose on social networks as salient. Hence, the individual may perceive an incoming notification as a welcome distraction and may react with increased attention towards this notification or further cues on social networks (e.g., the possibility to mention others in posts and to receive Likes). Thus, the subjective perception of a situation may be accompanied by specific affective and cognitive responses and subsequently, it can influence the triggering of a leading processing system and the resulting evaluation of possible options. This is in line with a recent study by Du et al. (2019), showing that an increased attention towards notifications (including perceived distraction frequency) can increase individuals’ failure to control the desire to use social media, which in turn could increase individuals’ self-disclosure tendency (see Walrave et al., 2012; Chang and Heo, 2014). In the depicted example, an individual’s lacking perception of possible negative consequences and their riskiness may be common in everyday life and may additionally lead to an increased self-disclosure tendency. The complexity of social networks and veiled negative (long-term) consequences may influence users’ perception in the way that they may not fully conceive the riskiness of self-disclosing, which in turn can influence their respective decisions. Consistently, other authors noted that possible negative (long-term) consequences are in principle not mentioned and thus not perceivable (Taddicken and Jers, 2011; Efroni et al., 2019), whereas social networks are primarily designed to fulfill their users’ needs and to let them experience immediate gratification (Taddicken and Jers, 2011).

Regarding the interplaying processing modes, cognitive control functions (such as inhibitory control) and the weighing of perceived short-/long-term benefits and risks associated with a specific disclosure decision (i.e., the core concept of the *Privacy Calculus Theory*, see Culnan and Armstrong, 1999; Dienlin and Metzger, 2016) can be allocated to the reflective system. In contrast, deciding based on gut feelings, heuristics, anticipated rewards, or urges would be attributed to the impulsive system (Bechara and Damasio, 2005; Schiebener and Brand, 2015). Due to the fact that social networks lack indications for strategic decisions (i.e., information and immediate feedback on possible negative consequences), which probably increases the relative degree of uncertainty of the respective decision situation, reflective processing can be complicated and the involvement of the impulsive system can be increased, similar to what is found for effects of stress on decision making in situations with increasing uncertainty (see Starcke and Brand, 2012). Furthermore, prior research also found that self-disclosures were related to neural responses ascribed to the impulsive system (including limbic structures). Tamir and Mitchell (2012), for instance, noted that self-disclosure may hold an intrinsic value due to the chance to introspect about oneself and to share this information with other people. This was supported by empirical data: neural regions that are associated with reward processing and part of the mesolimbic dopamine system (i.e., the nucleus accumbens, belonging to the ventral striatum, and the ventral tegmental area) have solidly been activated during self-disclosures (Tamir and Mitchell, 2012). This supports our critical questioning of the often proposed rational “calculus” for self-disclosure decisions, which would require especially prefrontal control. Actually, the decision to self-disclose can also be guided by the impulsive system since the respective situation may hinder cognitive control, which probably also explains other researchers’ remark that users often appear to be unhesitant when they share abundant information about themselves online (Tufekci, 2008; Kokolakis, 2017).

As noted above, individual and environmental features and resulting perceptions can impact the interplay of the inner systems so that individuals may decide rather reflectively or impulsively. For instance, knowledge about possible risks, which would actually support deliberation and controlled processes (reflective system), may be passed over by a strong anticipation of rewards and gratification, thus resulting in a shift towards impulsive processes as the leading mode. However, a high fear of risks (impulsive system) may also trigger the reflective system by exerting self-control and inhibition (see also Schiebener and Brand, 2015). Besides the interplay of the impulsive and reflective system, the interoceptive system is assumed to additionally interact with both systems. By processing bodily stages (e.g., in the form of sensing temptations resulting from an increased heart rate) it can be seen as a kind of mediator between the reflective and the impulsive system (Wood and Bechara, 2014), whereby it is expected to often lead to “the promotion of impulsive behaviors and the hijacking of decision-making processes concerned with the control of these impulses” (Turel and Bechara, 2017, p. 92; based on Naqvi and Bechara, 2010). In the case of self-disclosures on social networks, individuals could, for example, consciously experience urgent temptations and desires (e.g., the temptation to share a picture when being in a state of arousal) that augment an impulsive information processing and reduce possible reflective processes (e.g., the usage of knowledge about the probability of privacy risks). This interaction can then influence the evaluation of possible options (*level of evaluation*). The resulting evaluation processes can thereby take place very quickly (depending on the leading processing mode) and may also be dynamic and iterative (see also Cunningham et al., 2007; Van Bavel et al., 2012). For instance, an individual may evaluate, predominantly driven by the impulsive system, that sharing specific information is the favorable option, and thus (quickly) decides to self-disclose. However, this option might also be checked via the reflective system before the decision is made (e.g., deliberate consideration of potential risks), which illustrates that both systems may also continue to interact during the final evaluation. Further, additional information (e.g., negative or positive comments in other posts) can also be integrated, so that further options may be evaluated and the level of evaluation may be deepened.

The propositions presented in this section can help to better understand self-disclosure decisions on social networks. It is likely that the impulsive system is predominantly involved in many self-disclosure decision situations – potentially strengthened by bodily awareness – while reflective processes may be diminished. Even if research on a behavioral and neurocognitive level regarding such a tripartite decision-making model is generally scarce, it is notably progressing and thus underpins our effort to also apply such propositions to the area of self-disclosure on social networks. In this area, we are not aware of any work that has theoretically conceptualized such a tripartite structure of inner decision-making processes in detail yet.

### Self-disclosure decision, behavior-related consequences, and feedback loops

Following the evaluation of possible options, an individual is expected to make a respective *self-disclosure decision.* A person can, without further adjusting any privacy-related environmental aspects, decide to self-disclose with regard to different dimensions, including an informational and psychological dimension (see Burgoon, 1982), and with regard to, for instance, a certain depth or breadth (see also *Disclosure Decision Model* by Omarzu, 2000). For example, an individual could share very intimate information (e.g., feelings with regard to a new romantic relationship) within a post on social networks which is not restricted to a specific audience and can thus be seen and further shared by anyone. This case may arise if the impulsive system is triggered as the leading mode, for instance, if risks are mainly intangible and the individual has to rely on gut feelings and is led by anticipated short-term gratification, which is potentially fortified by bodily states (e.g., a fast heartbeat). Here, the interoceptive system could amplify impulsive processes and may alleviate reflective ones (see Turel and Bechara, 2016). Thus, the decision to share very intimate or extensive information without further adjustments may predominantly be led by the impulsive system that is potentially amplified by interoceptive processes. Apart from that, the decision to self-disclose without any privacy adjustments may also be the result of rather reflective processes, if an individual is, for example, quite aware of possible negative consequences and the lack of control over personal information, but strategically hazards the consequences in order to achieve specific long-term outcomes (e.g., increased popularity and impact on social networks). However, even if this might hold true for some users, we argue that most users are probably engaging in extensive self-disclosures on social networks due to the prospect of short-term rewards (such as positive feedback from friends), which appear more salient than possible negative long-term consequences and thus complicate reflective weighing processes.

Moreover, individuals may also decide to self-disclose (e.g., to a certain depth or valence) on condition that privacy-related environmental aspects are adjusted (i.e., preservative/corrective privacy regulations), for instance by changing the visibility of a post (see also *Privacy Process Model* by Dienlin, 2014; *Situational Privacy and Self-Disclosure* by Masur, 2018). Especially here, the proposed interaction between the different neural systems appears to be quite observable. Such a self-disclosure decision making is also in line with studies reporting that some users indeed restrict access to their information (on a horizontal level) by defining the audience, which is, however, not necessarily accompanied by lower self-disclosure levels (Stutzman et al., 2012, 2013). Further, a study by Wang et al. (2013) used different nudges including one that was meant to remind users of who would be able to see the post they are currently creating and the authors concluded that with this supporting information, users can be encouraged to (at least in some situations) change their privacy settings before posting. Thus, reflective processes may be triggered by such additional information and could lead, whilst also taking into account further intuitive or impulsive as well as interoceptive processes, to more deliberate self-disclosures.

Finally, individuals may also decide not to engage in self-disclosure on social networks. An individual may evaluate that self-disclosing would be too risky in a given situation (even though specific cues may also hint at potential benefits) and based on mainly reflective processes including monitoring and inhibitory control the individual decides not to self-disclose. Of course, it can also be argued that such decisions can as well be guided by the impulsive system – for example, if an individual is in general very risk-averse and perceives many situations as risky, thus also the respective self-disclosure decision situation, and the individual is then, for instance, led by strong emotions such as fear, which leads to the decision not to disclose. An individual could also generally (meaning in a rather automatic way based on past experiences and negative attitudes) not engage in self-disclosures across various situations. However, we argue that in many cases, the decision not to self-disclose on social networks is probably resulting from mainly reflective processes triggered by specific factors (e.g., the presence of profound skills or a warning message; e.g., Büchi et al., 2017; Ostendorf et al., 2022) which may lead to a correspondingly strong risk perception, although the impulsive system (and interoceptive processes) can be involved as well.

**Figure 3:**
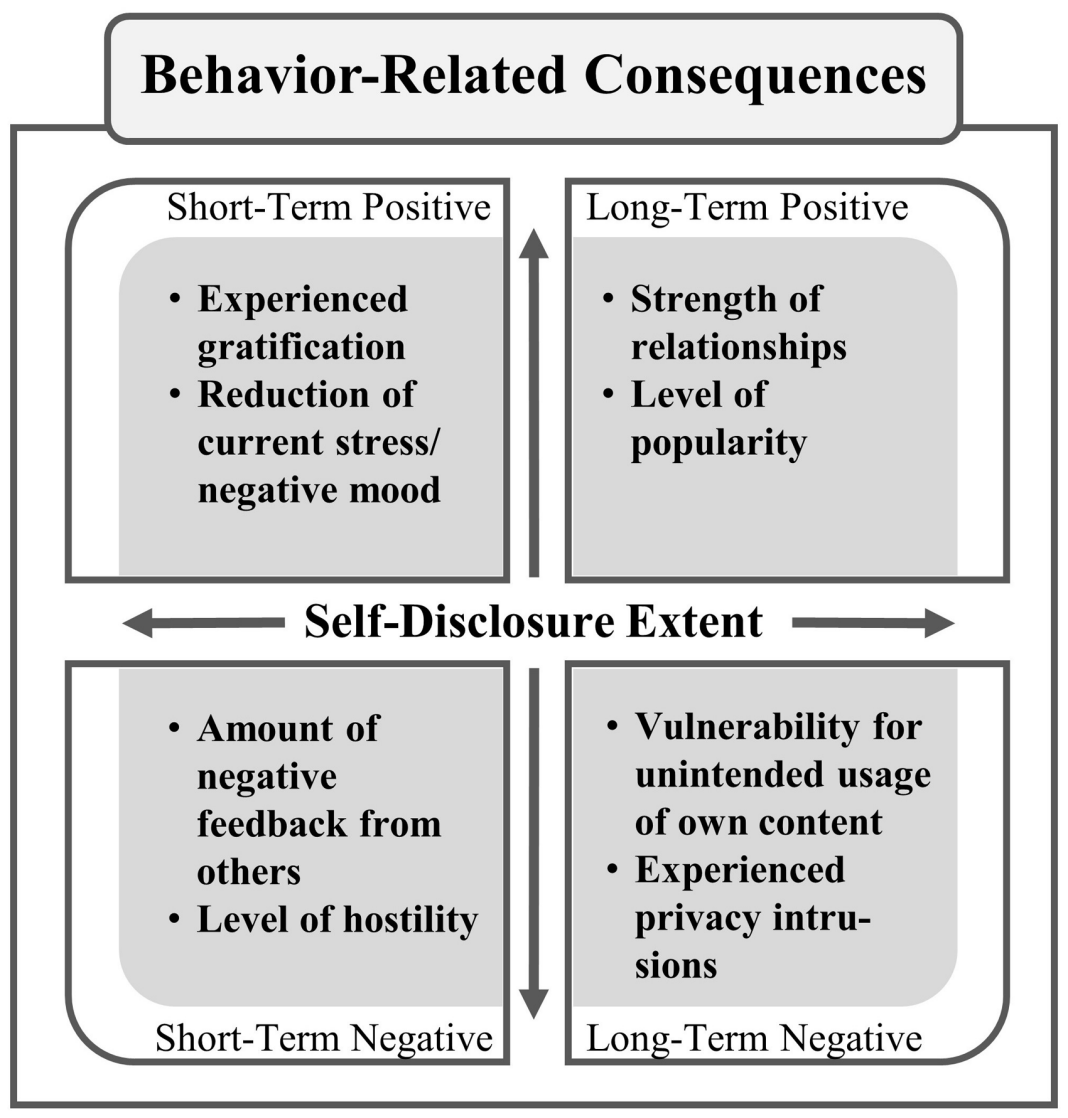
Classification of potential consequences related to the extent of individual’s self-disclosure on social networks (positive/ negative short- and long-term consequences can increase or decrease, depending on, for instance, the amount, duration, depth, or breadth of information disclosure). These potential consequences are exemplary and do not claim to be exhaustive.

After a self-disclosure decision has been made and individuals engaged in the respective behavior, various consequences can occur. These can be categorized as follows: *short-term positive*, *short-term negative*, *long-term positive*, and *long-term negative*. Depending on the self-disclosure extent, these consequences can also differ in their scope. An exemplary classification of possible consequences can be found in Figure 3. For instance, individuals may experience high immediate gratification or an increased reduction of negative mood (*short-term positive*) due to high self-disclosure on social networks, but may also in some situations experience an increased amount of negative reactions or dislike (*short-term negative*). In turn, both can - as a form of *feedback* - impact specific individual features (e.g., stabilizing individual’s reward sensitivity or broadening personal experiences), but also the inner decision-making processes in subsequent self-disclosure decision situations. It can further be argued that, for instance, the recurring experience of gratification due to individual’s self-disclosure on social networks could, in turn, lead to habitual disclosing behaviors that may automatically take place in specific situations. Following Schiebener and Brand (2015), risk-reinforcing feedback (e.g., experienced rewards) can promote a processing via the impulsive system, while risk-punishing feedback (e.g., negative comments from others) can promote a processing via the reflective system (see also Figner et al., 2009). Since risk-reinforcing feedback is probably most frequently experienced in the context of self-disclosures on social networks, a subsequent processing via the impulsive system (including an increased disregard of long-term consequences) may be amplified (*cf.* Schiebener and Brand, 2015; Müller et al., 2017), which can in turn be associated with the development of habituated and automatic behaviors (*cf.* Turel and Bechara, 2016). Moreover, the sensitivity towards specific cues (e.g., an application’s logo) as part of conditioning processes can be an important factor that may increasingly contribute to inconsiderate self-disclosing decisions on social networks (*cf.* Schiebener and Brand, 2015). This is also in line with research focusing on usage behaviors in media-rich environments (e.g., Naab and Schnauber, 2016; Hofmann et al., 2017; van Koningsbruggen et al., 2017, 2018). Further, certain *positive long-term* consequences (e.g., strengthened relationships) and *negative long-term* outcomes (e.g., increased vulnerability for unintended usage of personal content) due to extensive self-disclosures can also act as a form of positive or negative *feedback* (see also *Disclosure Processes Model* by Chaudoir and Fisher, 2010), whereby especially *negative long-term* effects may often not be recognized as being induced by the specific behavior. Thus, their impact on subsequent decisions may be lower than the impact of *short-term*, especially *short-term positive*, consequences. However, this still needs to be investigated more deeply in future studies. Overall, positive and negative short- and long-term consequences may also lead to the *initiation of subsequent environmental adjustments* (e.g., changing general privacy settings, deactivating push-notifications). For example, an individual may have posted a picture visible for the public (e.g., anyone on or off Facebook) and self-disclosed without modifying any environmental aspects. Subsequently, due to the experience of hostile comments, the individual may change default settings (e.g., who can comment their public posts) and further aspects (e.g., general profile visibility). On the contrary, experiencing positive comments and approval may also lead to a change from a previously limited post visibility (e.g., friends only) to the default of a public visibility for subsequent posting situations.

## Discussion and future directions

Research on causes and effects of individuals’ self-disclosures on social networks has been growing notably over the last years. However, there are still substantive gaps of knowledge, especially with regard to psychological mechanisms underlying self-disclosure decisions. The question of why so many people engage in extensive self-disclosures on social networks, although this can be accompanied with severe consequences, whereas others do not, has so far often been explained by proposing a rather strategic and calculative user. As highlighted here and by dual-and tripartite-system approaches that serve as a theoretical basis, such a view can be questioned since human decision making can also be guided by intuitive or impulsive processes. Furthermore, the perception and awareness of bodily stages (associated with the interoceptive system) may also be a relevant aspect that has not been investigated sufficiently by now, especially in the context of self-disclosure decision making on social networks. Consequently, previous findings that have been interpreted in light of the *Privacy Calculus* may need to be critically reinterpreted, especially since many studies did not directly provide evidence for a weighing process of benefits and risks (see also Knijnenburg et al., 2017; Dienlin et al., 2020). It therefore appears necessary to increasingly apply neuroimaging methods (e.g., functional magnetic resonance imaging, fMRI) when examining individuals’ self-disclosure decisions in order to receive a clearer picture of underlying mechanisms and involved neural systems based on respective brain activity. From a neuropsychological perspective, research should thus closer investigate the proposed involved systems, whereby especially a deeper exploration of the role of the insula and the interoceptive system may provide helpful new insights. However, it has to be taken into account that a specific construct or variable cannot be mapped clearly onto only one specific brain area and complex (impulsive, reflective, and interoceptive) processes involve multiple brain regions, which can be interconnected (see also Turel and Bechara, 2016). More research is still needed here to get a clearer picture of associations between behavioral and neural processes. Consequently, our proposed model is not meant to be final, but needs to be tested systematically in order to derive a better understanding of self-disclosure decisions - particularly in view of the fact that (a) there may of course be further noteworthy features that we do not explicitly mention in this manuscript, and (b) previous results are sometimes mixed (e.g., regarding the impact of individual or environmental features), as mentioned in the respective sections.

Nevertheless, we think that the TSDD model can strongly promote future research, especially due to the provision of theoretical assumptions regarding inner decision-making processes and also by outlining that specific constructs can interact with each other and thereby influence inner decision-making processes to different extends. This, as a whole, can help to disentangle seemingly paradoxical behaviors subsumed under the term *privacy paradox* (e.g., Barnes, 2006; Barth and de Jong, 2017). Future studies should therefore investigate interactions and interrelations between different features more systematically and also how these are associated with perception processes, neural activations, evaluation processes, and finally the respective decision. Systematic variations of the decision situation and experimentally manipulating different proposed features will help to better understand individuals’ decisions under different circumstances (see also Ostendorf et al., 2022). Moreover, by focusing on actual behavior rather than self-disclosure intentions, and by conducting longitudinal investigations by, for instance, applying ambulatory assessment tools, casual relationships can be better understood and protective approaches can be optimized. Interventions in the form of nudges or warning messages to prevent users from incautious information sharing online (by triggering reflective processes) could be optimized and also more systematically validated (i.e., which approaches work particularly well for which groups of people and under which circumstances). The general need for user support is also highlighted in several recent works (e.g., Acquisti et al., 2017; Spottswood and Hancock, 2017; Aïmeur et al., 2019; Díaz Ferreyra et al., 2019, 2022; Efroni et al., 2019; Krämer and Schäwel, 2020; Meier et al., 2020) and protective approaches likely need to be dynamic in order to react to dynamic changes within the user and the environment. With our suggested process model, we can aid in facing such challenges (e.g., what kind of warning message supports whom in what situation?) and in solving further remaining research questions. The TSDD model enables researchers from different disciplines to formulate concrete hypotheses of interest including moderation and mediation hypotheses on potentially preventive features and factors that may complicate the triggering of reflective processes as well as more complex hypotheses (e.g., addressed using structural equation modeling) on the involvement of the proposed neural systems. One example may be: The positive relationship between trait impulsivity and the extent of shared information on social networks can be reduced by the presentation of warning messages. Researchers may also be interested in more complex hypotheses including interoceptive processes, for example: With increasing temptation to use social networks (indicating the triggering of the interoceptive system), people share an increased amount of information on social networks; the temptation to use social networks further strengthens the positive effect of trait impulsivity (expected to trigger the impulsive system) and weakens the negative effect of self-control (expected to trigger the reflective system) on the amount of shared information on social networks. In order to test such hypotheses, researchers can apply different measurements and experimental designs.

As exemplary specifications of the more generic hypotheses just mentioned, researchers could derive the following assumptions regarding the first one: Given that the motor impulsivity facet (e.g., to act on impulse) was found to be positively related to the extent of online information disclosure (Aivazpour and Rao, 2020), this relationship is also likely observable in the specific context of social networks. Individuals with high motor impulsivity may lack inhibitory control that is necessary to counteract or suppress rather spontaneous and reflexive reactions to those many input and interaction possibilities provided on social networks (Aivazpour and Rao, 2020), which may result in broad self-disclosures. Besides, research has shown that warning messages with information on potential negative consequences can support more deliberate self-disclosure decisions, thus likely triggering reflective processes. For instance, for individuals who received a warning message on a fictitious social network that informed about possible short- and long-term negative consequences, the likelihood of creating a post (rather than not creating a post) was lower compared to those who did not receive a warning message (Ostendorf et al., 2022). For those who proceeded creating a post, however, the presentation of this warning message did not reduce the amount of shared information within the post, thus calling for more research. Specified and more dynamic warning messages may be helpful for individuals with certain characteristics, such as high levels of motor impulsivity. Receiving content-dependent warning messages in temporal proximity to information sharing decisions (i.e., popping up when clicking on the button ‘post’) may support those individuals by triggering reflective processes and reducing impulsive ones. For such research purposes, specific tools (e.g., the app ENAGRAM as introduced by Díaz Ferreyra et al., 2022) may be used and adapted in order to manipulate experimental conditions (e.g., post-dependent warning messages versus no warnings) and to collect and analyze behavioral data (e.g., by implementing content analysis techniques into ENAGRAM, the breadth of users’ disclosures via posts could be assessed). Thus, a specified hypothesis may be: The positive relationship between motor impulsivity (assessed with the Barratt Impulsiveness Scale, BIS-15, Spinella, 2007) and the breadth of shared information via posts (measured with an adapted version of the app ENAGRAM by Díaz Ferreyra et al., 2022) is reduced by the presentation of warning messages with post-dependent information on possible short- and long-term consequences.

Regarding the more complex hypothesis, researchers may derive the following specification: With increasing temptation to use social networks in boredom/idle time situations (measured with items proposed by Turel and Bechara, 2016), people share an increased breadth of information on social networks (measured with a modified version of the Revised Self-Disclosure Scale, see Hollenbaugh and Ferris, 2014); the temptation to use social networks in boredom/idle time situations further strengthens the positive effect of motor impulsivity (assessed with the BIS-15, Spinella, 2007) and weakens the negative effect of self-control (assessed with the Self-Control Scale, Tangney et al., 2004) on the breadth of shared information on social networks. As highlighted in previous work, boredom or idle time may be a key driver of social networks usage by creating a strong sense of temptation (Turel and Bechara, 2016). Social networks usage is further positively associated with the tendency toward self-disclosure (e.g., Walrave et al., 2012), so that the temptation to use social networks in boredom/idle time situations likely increases individuals’ extent of information sharing. Further, this temptation may perturb the balance between impulsive and reflective processes, so that it strengthens the involvement of impulsive ones and reduces the involvement of reflective ones (see also Turel and Bechara, 2016). As argued above, individuals with increasing levels of motor impulsivity may lack necessary inhibitory control and thus share broader information about themselves on social networks. This relationship may be further amplified by temptation in boredom/idle time situations. In contrast, an increased ability for self-control can be a buffering factor and was found to be related to lower self-disclosure on Facebook (Yu, 2014). Thus, for individuals with higher levels of self-control, the reflective system may predominantly guide decision making, which may manifest in a reduced breadth of shared information. However, strong temptation in boredom/idle time situations may impair the involvement of the reflective system so that the effect of self-control is weakened. Finally, in addition to deriving various hypotheses from the TSDD model and specifying them, as outlined here by way of example, future research may also build upon our propositions and embed them into a wider framework which also considers a group level of information sharing (see, for instance, the theory of multilevel information privacy by Bélanger and James, 2020).

## Conclusion

The Tripartite Self-Disclosure Decision (TSDD) model is a theoretical framework developed to explain and conceptualize individuals’ self-disclosure decisions on social networks. It contains different components, whereby individual and environmental features are proposed to (interactively) influence inner decision-making processes that subsequently lead to the final behavioral decision and corresponding short- and long-term consequences, which, in turn, can reinforce certain components in subsequent decision situations. Subjective perception processes, the interplay of three neural systems, and evaluation processes are thereby components of the inner decision-making processes. A reflective, an impulsive, as well as an interoceptive system are considered as important interplaying neural systems. Based on dual-and tripartite-system perspectives and current progression in neurocognitive research on decision-making, this model focuses on potential mechanisms underlying the decision to self-disclose on social networks, which may result in negative consequences including privacy breaches. By taking such a neurocognitive perspective, we expand current research and existing models on privacy-related decisions online. Although the TSDD model is already based on theoretical assumptions and empirical results from different disciplines, its hypothesized constructs and processes should be examined systematically in future studies. This will help to better understand seemingly paradoxical behaviors and may clear up why individuals provide large amounts of personal information on social networks, although this can be accompanied by severe negative consequences on various levels. Finally, this model could also help to develop more effective support measures that prevent users from incautious information sharing once the underlying and complex mechanisms have been examined in greater detail.

## Data availability statement

All original contributions are included in the article, further inquiries can be directed to the corresponding author.

## Author contributions

All authors contributed to conceptualization and visualization. SO wrote the first draft of the manuscript. MB supervised the work and reviewed and edited the manuscript. All authors reviewed and commented on previous versions of the manuscript. All authors contributed to the article and approved the submitted version.

## Funding

This work was supported by the German Research Foundation (DFG) under grant no. [GRK 2167] Research Training Group “User-Centred Social Media.” We acknowledge support by the Open Access Publication Fund of the University of Duisburg–Essen.

## Conflict of interest

The authors declare that the research was conducted in the absence of any commercial or financial relationships that could be construed as a potential conflict of interest.

## Publisher’s note

All claims expressed in this article are solely those of the authors and do not necessarily represent those of their affiliated organizations, or those of the publisher, the editors and the reviewers. Any product that may be evaluated in this article, or claim that may be made by its manufacturer, is not guaranteed or endorsed by the publisher.
